# Typification and Characterization of Different Livestock Production Systems of Mediterranean Dairy Sheep Farms with Different Degrees of Intensification: A Comparative Study

**DOI:** 10.3390/ani15030448

**Published:** 2025-02-06

**Authors:** Manuel Gonzalez-Ronquillo, Lizbeth E. Robles-Jimenez, Jorge Osorio Avalos, Isabel Revilla, Cristina Hidalgo-González, Pilar Rodriguez, Jaime Nieto, Javier Plaza, Carlos Palacios Riocerezo

**Affiliations:** 1 Facultad de Medicina Veterinaria y Zootecnia, Instituto Literario 100, Universidad Autonoma del Estado de Mexico, Toluca 50000, Mexico; lizroblez@hotmail.com (L.E.R.-J.); josorioa@uaemex.mx (J.O.A.); 2 Escuela Politécnica Superior de Zamora, Universidad de Salamanca, Av. de Requejo, 33, 49029 Zamora, Spain; irevilla@usal.es; 3 Facultad de Ciencias Económicas y Empresariales, Universidad de León, Joaquín Gonzalez Vecín, 24007 Leon, Spain; cristina.hidalgo@unileon.es (C.H.-G.); pilar.rodriguez@unileon.es (P.R.); 4 Facutad de Ciencias Agrarias y Ambientales, Universidad de Salamanca, Av. de Filiberto Villalobos, 119, 37007 Salamanca, Spain; jaimenl@usal.es (J.N.); pmjavier@usal.es (J.P.)

**Keywords:** production systems, sheep, typification, milk, NRMS, MESMIS

## Abstract

It is necessary to study small-ruminant production systems in a spatial context, as different areas, depending on their natural, socioeconomic, and cultural context, will respond differently to the effects of such causal factors. The aim of this study was to analyze the relationships between technology use and farm and farmer characteristics in dairy sheep production by developing a farm typology using multivariate statistical analysis and comparing it with the multi-scale sustainability evaluation of the Natural Resource Management Systems (NRMS) method. Quantitative and qualitative data were evaluated and compared using the NRMS method and cluster analysis to identify groups of farms. Fourteen variables were considered for the analysis, resulting in four farm systems with the NRMS method, named Intensive, Semi-Intensive, Semi-Extensive, and Extensive; however, when analyzing the data by cluster analysis, the following clusters were used: Cluster 1, Semi-Extensive Dairy Systems (SEDSs); Cluster 2, Semi-Intensive Systems (SIDSs); Cluster 3, Semi-Extensive Cheese Systems (SECSs); and Cluster 4, Intensive Dairy Systems (IDSs). This study concludes that the traditional classification of dairy sheep farms based on their dependence on natural resources can be enriched by incorporating additional indicators that allow for a more comprehensive and accurate characterization of farms.

## Simple Summary

The relationships between technology use and farm and farmer characteristics in dairy sheep production were studied across 70 dairy sheep farms. Five factors were identified: F1, milk production (23.8%); F2, animal species in the flock (18.1%); F3, product transformation (cheese) (14.2%); F4, energy use (8.8%); and F5, hectares per animal (7.2%). Four different clusters were then identified: Semi-Extensive Dairy (SED), Semi-Intensive (SI), Semi-Extensive Cheese (SEC), and Intensive (I) Systems. The traditional classification of dairy sheep farms according to their dependence on natural resources can be complemented by the inclusion of other indicators that allow for a more complete differentiation of farms.

## 1. Introduction

Extension and development policies have traditionally focused on the average farmer, without considering the socioeconomic and environmental contexts in which they live [1]. Animal production systems in small ruminants (sheep and goats) have different characteristics. To understand these systems, it is necessary to develop a guide and an objective on development and extension policies [2,3]. The characterization of studies is usually based on the description of production systems with the available resources and their management, but it is necessary to understand the complexity of the relationships between the resources of the farms and the capacity of the farmers, which is specific due to social conditions, access to information, and intensity of management; it can also be defined by the number of production technologies and the frequency of its use on farms [4].

The “systems approach” in agricultural research and extension considers an agricultural system as the set of decisions related to household production and consumption. This includes aspects such as crop selection, livestock rearing, off-farm activities, and household consumption [5]. However, each farm household faces unique situations due to the diversity of resources and issues it faces, resulting in distinct farming systems. Given the impracticality of addressing each case individually, farms are grouped into “recommendation areas”—clusters of farmers with similar conditions who can benefit from shared recommendations. For this classification to be effective, it must maximize homogeneity within each group and heterogeneity between them. However, methodologies such as surveys or rapid rural appraisals tend to associate recommendation domains with specific geographical areas, overlooking the significant diversity within each area [6].

In order to assess the sustainable development of farms, the use of the three dimensions of sustainable development has been considered using the 3Ps (people, planet, and profitability), because a synergy of these different implications is sought; it has been commonly accepted that sustainable development consists of three dimensions: social security (people), environmental responsibility (planet), and economic efficiency (profitability) [7].

Sustainable development calls for a long-term structural strategy for the world’s economic and social systems, which aims to reduce the burden on the environment and natural resources to a permanently viable level, while maintaining economic growth and social cohesion, allowing it to be sustainable in the long term.

Therefore, it is necessary to study the production systems in small ruminants in a spatial context, since different areas depending on their natural, socioeconomic, and cultural context will respond differently to the effects of such causal factors; the diversity of systems will be better described by an integrated group of spatial characteristics and variables [8]. Each production system is a particular system, and therefore, one might think that it is ideal to have an individual intervention strategy for each one. However, this individualized planning is hardly viable from an economic and institutional point of view. For this reason, the development of taxonomies in production systems has been proposed, enabling the grouping of similar farms to design more effective tools and policies [9].

The criteria used to create typologies depend largely on the objectives for which they are constructed [6]. Some classification criteria are based on finding the indirect factors that lead owners to make certain decisions [10] or explaining the productive strategies that owners have developed or have the potential to develop [11].

Castilla and León Spain face a common problem in creating typical representations of farming systems. The present study utilized data in order to develop mathematical programming decision-making models for typical farming systems. The hypothesis of the present study was that the construction of taxonomies in dairy sheep production systems, based on criteria that identify indirect factors and productive strategies, allows for the grouping of farms with similar characteristics, facilitating design and decision making to improve their productivity. The objective of this study was to analyze the relationships between technology use and farm and farmer characteristics in dairy sheep production by developing a farm typology using multivariate statistical analysis and comparing it with the indicator-based Framework for the Evaluation of Natural Resource Management Systems (NRMS) method.

## 2. Materials and Methods

### 2.1. Study Location and Sampling Strategy

The study area corresponds to the geographical area of Castilla y Leon, where the activities carried out in small-ruminant milk production systems are based on the use of natural community resources, mainly in the agricultural and forestry sector and, in other cases, are based on the use of animals housed with the purchase of concentrates and their sale as liquid raw milk or their transformation into cheese.

**Figure 1:**
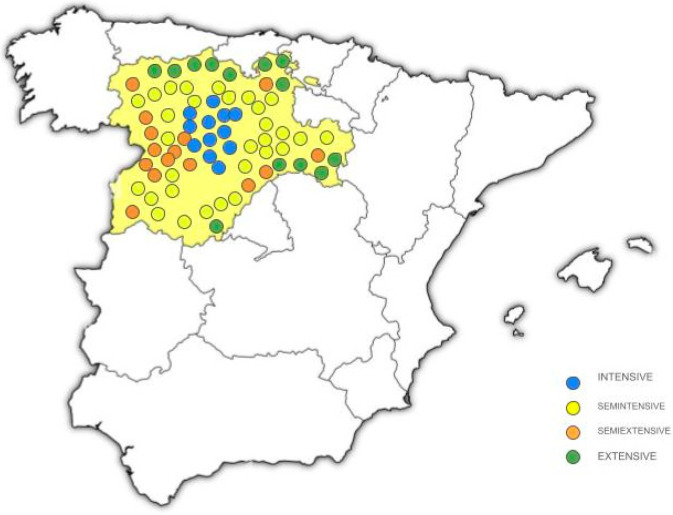
Map showing the locations of the 70 different dairy farms in the region of Castilla y León, Spain. Blue, Intensive (I) production system (*n* = 12); Yellow, Semi-Intensive (SI) system (*n* = 30); Orange, Semi-Extensive (SE) production system (*n* = 14); Green, Extensive (E) system (*n* = 14).

This study was carried out on a random sample of rural livestock farmers (Table 1, Figure 1), considering 70 dairy sheep farms, of which 12 farms had an Intensive production (I) system according to the traditional classification [9], without the animals going outside. Thirty farms had a Semi-Intensive (SI) system, using natural resources to some extent, both by bringing them closer to confined animals and by using them for non-productive animals. Fourteen farms had a Semi-Extensive (SE) production system, in which animals are fed mainly natural resources and are supplemented with feed from the farm itself, including their young in times of scarcity. Once the lambs are weaned, they continue to remain in the facilities, and the rest of the animals graze on natural resources. Fourteen farms had an Extensive (E) system, where the external input of resources is minimal and animals spend maximal time outdoors and consuming natural resources.

Farms were classified using the traditional criteria presented by Hidalgo-González et al. [12]. The sampling method used was the two-stage cluster method. The selected farmers were visited periodically every month by project collaborators. Data were collected regarding the ownership of the farm, the age of the owners and workers, their work structure, the livestock census, and the number of hours and days that the animals graze during the year. From the agricultural census, the following data were collected: the types of crops, the area cultivated or used by the animals, and the type of management they carry out. Additional data included the following: the number of installations and machinery they use, the purchases and sales made, reviews of invoices, consigning quantities, and price of each concept. These data were collected over a three-year period by two people who visited the farms. Fuel and electricity consumption was recorded and accounted for using purchase invoices and delivery notes. In addition, both salaried and family labor were included in the calculation of the labor force. As a result, a total of 124 indicators were established, distributed among 27 economic indicators, 46 environmental indicators, and 65 social indicators. Subsequently, the project’s researchers, livestock farmers, and agents in the sector met to try to define 14 indicators that, according to their criteria, were more representative of a defined production system (Table 2).

The NRMS method highlights the importance of the local factor in the diagnosis by integrating internal responses, making it a continuously evolving tool [13]. This assessment has a comparative and cyclical character, which starts with the characterization of the system, followed by the integration of indicators, and ends with the formulation of conclusions and recommendations to optimize management. The main phases of the NRMS include [13] definition and description of the farms studied, characterization of the systems, selection of indicators and development of attributes, overall assessment of the tools and measurement of sustainability, as well as the writing of a proposal. The NRMS method considers the local factor as a key component of the diagnosis, which allows for the identification of endogenous responses and positions it as a constantly evolving tool. The assessment under this approach should be comparative and cyclical, starting with the characterization of the system, followed by the integration of indicators, and culminating in the formulation of conclusions and recommendations aimed at optimizing the management of the system.

### 2.2. Typification of Production Systems

In this study (Table 1), 14 variables were used, selected by the working group, which were as follows:

a. Within the structure of the farm:
  1. Herd size (number of sheep);
  2. Births per year (how many births per year);
  3. kg of milk per sheep per year (kg of milk/year/animal);
  4. kg of concentrate purchased per animal per year;
  5. Animals in housing (yes/no).
b. From the management of the agricultural area:
  6. Hectares per useful agricultural area per ewe (Ha AAS/ewe), which is the result of subtracting the total area of pasture, as well as the non-agricultural area (areas occupied by forest species, uncultivated land, or land occupied by bushes) and the total area of dry or irrigated land with crops not used by the livestock [14];
  7. Community days per animal per year, which is the time that an animal spends on communal land (areas that provide pasture, fertilizer, and firewood, among other necessary resources), belonging to a given population, which covers basic subsistence needs thanks to these resources (pasture and shelter) [15].
c. Environmental indicators (planet):
  8. Electricity consumption per animal and year;
  9. Fuel use per sheep and year (liters);
  10. Number of animal species present (i.e., goats, cows, donkeys, and horses)—this variable considered only one or more species present on the farm [16];
  11. Percentage of native animals of the total herd (i.e., number of animals present on the farm)—the breeds used in the present study were Churra, Castellana, Assaf, Lacaune, and crosses among them;
  12. kg of minerals that have been added to the soil.
d. Social indicators, such as the following:
  13. Whether there is transformation into cheese (yes/no);
  14. The relationship between the family work unit and work unit (FWU/WU). One annual work unit is equivalent to one person employed full-time on a farm [16]. Persons who do not work the whole year on the operation constitute a fraction of the annual labor work unit (ALWU); one ALWU is equivalent to 1826 h/year [17]. The number of family workers of the total workers (FWU/WU) is the labor force of a family unit; family workers are responsible for generating the family income [18,19].

The average indicators for the initial groups of farms can be seen in Table 1. Significant differences (*p* < 0.05) were found between the groups in the most defining indicators of the systems, such as herd size, number of birth groups, milk production, presence of animals on the premises or use of natural grazing land, kilos of concentrate consumed, and use of indigenous breeds. However, several indicators were not definitive among the groups of farms. The statistical differences between the different systems do not follow the same pattern in the indicators studied; this can be seen in the different-letter super indexes a, b, and c. We only found a statistical definition between each group in the indicator for milk production per sheep, where the most productive are the Intensive farms and the least productive are the Extensive ones, while the Semi-Intensive ones are in the middle. The same is true for the use of indigenous breeds, which are more linked to Extensive systems and less so to highly specialized ones.

The indicators selected do not fully define the farms used in this work, so we were forced to carry out other multifactorial analyses to find typical indicators for the farms.

### 2.3. Classification and Characterization of Production Systems

The information obtained should be reviewed to eliminate variables with no variability before performing the factor analysis. It is important to discard those variables, whose low variability does not contribute to the distance measure used to form clusters [6]. Furthermore, if certain variables are not relevant to the specific objectives of a study, they can be eliminated, even if they initially appear consistent with the observed farming systems [20]. Another point to consider is whether highly correlated variables should be excluded, as their use without critical analysis implies an implicit weighting that could distort the results [21]. In these cases, the influence of one correlated variable is already reflected in the changes in another variable. Finally, cluster analysis requires addressing missing data, and this can be performed either by discarding incomplete observations or by replacing missing values with averages. However, in these cases, the sample size could be reduced, or biases could be generated. Therefore, it is preferable to eliminate the affected variables rather than the observations.

### 2.4. Statistical Analysis

The analysis of the variables described was carried out in different independent stages. With the information of the 14 variables obtained from a survey of the 70 production units, an analysis of the interrelations between them was made, and their contribution to the total variance that existed between the herds that participated in this study was estimated (multivariate analysis). Subsequently, a factorial analysis and a correlation analysis were performed using the PROC FACTOR of the statistical software SAS 9.2 (SAS Institute Inc., 2007, Cary, NC, USA). The variables with a commonality (h < 0.9) were not included in the factor analysis, indicating that these variables were not correlated with the new factors. The factors selected were those with eigenvalues ≥1. In order to obtain a better understanding of the factors obtained, an orthogonal rotation method (varimax) was used, so that the factor values in the analysis were estimated by the regression method and saved as new variables. The grouping of herds in homogeneous groups was carried out through SAS’s hierarchical clustering procedure, PROC CLUSTER using the variables described above.

An analysis was then carried out to confirm if the clusters between the herds were different (Tukey test, *p* < 0.05) for each of the variables considered in this study among the conformed clusters and to determine if there were significant differences. After obtaining groups of flocks with similar productive conditions, descriptions by cluster were made from the variables considered for this study.

## 3. Results

### 3.1. Multivariate Analysis

Factorial Analysis: For the first part of this study, 14 variables were considered for the analysis, resulting in five vectors that explained 72.1% of the total variance between herds (Table 3).

Factor 1, called milk production, represented the largest contribution (23.8% of the total variance between farms), comprised variables related to the degree of technification, as follows: kg milk/sheep/year, standard milk, kg of concentrate purchased per animal per year, housing, communal days/animal per year, and percentage of native animals of the total herd. Factor 2, called animal species in the herd, which represented the second largest contribution (18.1%), was considered in general those variables that involving the species present in the herd, i.e., the number of animal species present in the herd. Factor 3, called product transformation, which predominantly consisted of variables that refer in general to the herd and its transformation of products, contributed 14.2% of the total variance between the farms, these were as follows: size of the herd or number of animals, births per year, if there is transformation of cheese, and the family work unit relationship (FWU/WU). Factor 4, called energy use, represented 8.8% of the total variance in the use of energy: electricity/animal intake per year, use of fuel/sheep per year and kilograms of minerals/ha added to the land. Factor 5, called hectares for livestock, corresponding to a single variable on the area in hectares for livestock (hectares per useful agricultural area per sheep) and hectares per useful agricultural area per sheep (AAS ha/animal), represented 7.2% of the total variance.

### 3.2. Cluster Analyses

**Figure 2:**
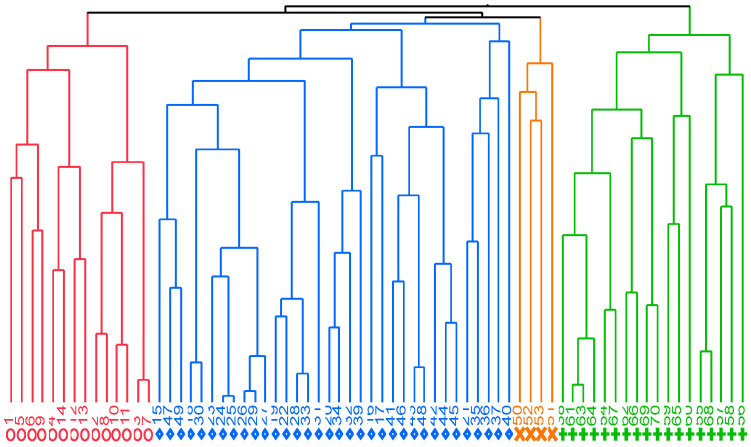
Clusters of the different dairy sheep production systems in northern Spain. CL1: Semi-Extensive Dairy Systems (red lines), CL2: Semi-Intensive Systems (blue lines), CL3: Semi-Extensive Cheese Systems (orange lines), CL4: Intensive Systems (green lines).

Four different clusters were formed based on the structural characteristics and economic results of the farms (Table 4, Figure 2): The first cluster (CL1, red lines), called Semi-Extensive Dairy Systems (SEDSs), includes 14 production units (PUs) and is characterized by having the smallest herd size and a lower reproductive intensity owing to fewer lambing periods per year. The animals are grazed only a few days per year, with low energy use (electricity consumption/animal per year and fuel/sheep per year). The transformation of milk into cheese is minimal. These PUs have the highest number of animal species present and one of the highest percentages of native animals in the total herd, which in turn have a minimal number of housed animals. Likewise, they register the production of kg of milk/sheep/year, supported by having the highest availability of hectares of useful agricultural area per animal (using the highest amount of kg of minerals/ha added to the land) and the highest amount of concentrate purchased per animal per year. They are mixed farms with significant agricultural activity aimed at providing resources to the animals. In terms of the initially proposed classification of farms, only 28% of them obtained the same classification, while 57% of the farms in this cluster are classified as Semi-Intensive, i.e., “Semi-Extensive Dairy Systems”; that is because, in terms of the variables associated with these farms, they have the smallest herd sizes, small numbers of births per year, and low use of energy (electric/animal consumption per year and fuel/sheep per year). The second (CL2, blue lines), called “Semi-Intensive Systems”, is characterized by having a small herd size, the lowest number of births per year, and high use of energy (electric/animal consumption per year and fuel/sheep per year). They have low milk control and a minimal transformation of milk into cheese. In the third cluster (CL3, orange lines), called “Semi-Extensive Cheese Systems”, we can find those with the largest livestock inventory, a greater use of energy (electricity/animal consumption per year and fuel/sheep per year), and adequate milk control. The fourth cluster (CL4, green lines) is called “Intensive Systems” and had the lowest number of communal/animal days per year; there is no transformation of milk into cheese.

Cluster 2, called Semi-Intensive Dairy Systems (SIDSs), comprised the largest number of PUs (n = 34), was characterized by having small herd sizes and the lowest rates of lambing per year. In this cluster, it was recorded that the animals had a reduced number of communal/animal days per year. This group of PUs showed that they had a high use of energy (electric/animal consumption per year and fuel/sheep or goat per year). They have low milk control and a minimal transformation of milk into cheese. These PUs have the lowest number of animal species present and the highest percentage of indigenous animals in the total herd, with animals partially kept in stalls. In these PUs, intermediate values in the production of kg milk/sheep/year were recorded, not having a significant number of hectares per useful agricultural area per animal (using a low amount of kg of minerals/ha added to the land) and with limited use of concentrate purchased per animal per year. In terms of the initially proposed classification of farms, only 38% of them obtained the same classification, while 41% of the farms in this cluster were classified as Extensive.

Cluster 3, called Semi-Extensive Cheese Systems (SECSs), formed by the smallest number of PUs (n = 4), was characterized by having the largest inventory of sheep and the highest number of births per year of the four clusters. This cluster registered the highest number of communal days/animals per year. This group of PUs showed a higher use of energy (electricity/animal consumption per year and fuel/sheep per year) due to the needs of milk processing. They have adequate dairy control. The flocks that make up this grouping have a minimal number of animal species present and the highest percentage of indigenous animals of the total flock that partially use animal housing. They have the lowest production of kg of milk/ewe/year, have a limited number of hectares of useful agricultural area per animal (also using a limited amount of kg of minerals/ha added to the land) and the lowest contribution of concentrate purchased per animal per year. In terms of the initially proposed farm classification, 75% of the farms obtained the same classification, while 25% of the farms in this cluster were classified as Semi-Extensive.

Cluster 4, called Intensive Dairy Systems (IDSs), comprising 18 PUs, was characterized by having an substantial inventory in the number of animals and a greater rate of lambing per year. The animals were recorded to have the lowest number of communal/animal days per year. In this cluster, the herds’ use of energy was substantial (electric/animal consumption per year and fuel/sheep per year). Dairy control is generally implemented in these PUs, and there is no transformation of milk into cheese. These PUs present a minimal number of animal species present, and these flocks have the lowest percentage of native animals of the whole herd, while having the highest number of housed animals with respect to the other three clusters. Likewise, the cluster recorded the highest record of kg of milk production/sheep/year, supported perhaps by the highest contribution of concentrate purchased per animal per year, with respect to the other clusters. The PUs that make up this cluster have the least availability of hectares per useful agricultural area per animal (using the least amount of kg of minerals/ha added to the land). The variable family work unit ratio (FWU) did not show differences (*p* < 0.05) among the four clusters. In terms of the initially proposed classification of farms, 50% of them obtained the same classification, while the other half of the farms in this cluster were classified as Semi-Intensive.

## 4. Discussion

Our survey methodology and statistical analysis helped us to typify and characterize different production systems of Mediterranean dairy sheep farms. The data obtained should enable and help to develop production capacities for small-ruminant farmers by integrating scientific knowledge with local knowledge [22].

### 4.1. Multivariate Analysis

Factor 1 (Table 3) explains 24% of the overall variance, and the variables were related to the degree of technology adoption and systematization. Farms represented by this factor might be those of small-scale producers that use their animals as a dietary protein source or producers wanting to supplement their income for the household. This situation is similar to what has been reported in dry areas [23] where very few Intensive systems are found, especially for milk production by small ruminants.

Other factors found in the multivariate analysis are related to the number of animal species within the herd (Factor 2), the processing of food such as cheese (Factor 3), energy input into the system (Factor 4), and land-surface use for small-ruminant production (Factor 5). All these factors together represent at different levels the most important challenges for small-ruminant producers, as they can be related to the efficient use of natural resources, the efficiency of feeding and flock management, and possibly the feasibility of marketing small-ruminant products.

### 4.2. Cluster Analyses

Under the conditions of this study, the farms from Cluster 2 represent the larger number of surveyed farms. They are characterized by small herds, which use low energy input and minimal control of milk quality and/or processing in their production systems. This group of farms seems to reflect the global situation of small-ruminant production systems. In some cases, small-ruminant productions have been largely excluded from organized markets and have not followed the same paths of development and specialization compared to other types of animal production such as large-ruminant, poultry, and swine production [24].

Cluster 2 shows situations where farmers are not well connected with the market chain and still do not want to consider technological implementations that comply with the existing regulations or/and anticipate regulations to be developed by community and local quality/health control agencies [25].

Small-ruminant production systems are characterized by low-input and low-output systems, and these types of systems are often related to social variables such as low education [24], which leads to reliance on traditional systems rather than the adoption of new professionalized systems. This probably explains to some degree, for example, why goat farming has not been successful in Mediterranean systems such as those from Spain, Italy, or Greece where there is irregularity in the available resources and the complexity of human resources in valorizing them [23]. The results from this study reveal the need to focus on the following research areas: (1) value-adding with an emphasis on product safety and quality and product naturalness, (2) assessment of production opportunities and constraints, (3) assessment of the management of animal nutrition to align breed selection with the market and the available natural resources, and lastly, (4) linking development and policies to upscale research achievements.

Future studies in this region of Spain and Mediterranean area should consider how to improve animal nutrition and breeding management as well as examine the state of basic training/education on market-oriented technologies.

Regarding nutritional management, the following are recommended: the use of innovative technologies targeted at increasing feed resource availability, rumen manipulation using natural compounds to boost microbial activity, improvement in diet quality, alleviation of feeding costs, and better control of livestock watering [26].

Also, small-ruminant production should be regarded as a source of multiple products: milk, meat, fiber, and skin [27]. Usually, farmers are unaware of the wide variety of products that can be obtained from their animals, and therefore, non-breed specialization can be found in Factor 1 and Clusters 2 and 3.

The animal sciences together with social sciences have the challenge of upscaling research for the benefit of small-ruminant farmers and their communities. Usually, small-ruminant farmers are conservative in their choice of strategies in coping with a productive variable and are reluctant to change their practices. However, farmers do gradually express interest in market-oriented technologies and then venture into production intensification and diversification [28].

Further research should be carried out on the classification and characterization of production systems, where the main limitations of these studies are the quality of the data collected and the rigor of the decision making.

## 5. Conclusions

The relationships between the use of technology and the characteristics of both farms and farmers involved in dairy sheep production in Castilla y León (Spain) were analyzed. Five main factors explain 72.1% of the total variability between flocks: milk production (23.8%), animal species in the flock (18.1%), product transformation (14.2%), energy use (8.8%), and livestock area (7.2%). Based on the structural characteristics and economic performance of the farms, four distinct groups were established. The traditional classification of dairy sheep farms based on their dependence on natural resources could be complemented by integrating other indicators that allow for a more exhaustive and precise characterization. These results could help dairy farmers to define which production system they are in, so that they can better adapt to the characteristics of each type of system.

## Figures and Tables

**Table 1:** Characteristics of the dairy sheep production unit in “Castilla y León” in Spain according to Hidalgo-González et al. [12], using the principal 14 variables.

	Intensive	Semi-Intensive	Semi-Extensive	Extensive	*p*-Value
N	12	30	14	14	
Number of sheep	681.3 ± 402.4 ^a^	483.9 ± 240.4 ^ab^	473.2 ± 347.7 ^b^	353.5 ± 348.0 ^b^	0.0310
Lambing per year	3.00 ± 0.7	1.96 ± 0.8 ^b^	1.67 ± 1.1 ^b^	1.67 ± 1.1 ^b^	0.0010
Milk per sheep (kg)	331.4 ± 137.1 ^a^	227.5 ± 90.8 ^b^	131.75 ± 78.0 ^c^	131.7 ± 78.0 ^c^	0.0001
Animals in housing (%)	67.9 ± 42.9 ^a^	41.2 ± 33.1 ^b^	31.7 ± 19.3 ^b^	31.7 ± 19.3 ^b^	0.0010
Ha AAS/sheep	0.07 ± 0.07	0.23 ± 0.24	0.18 ± 0.20	0.18 ± 0.20	0.0700
Communal land use (days)	0.0 ± 0.0 ^b^	20.2 ± 43.6 ^b^	101.0 ± 68.5 ^a^	101.0 ± 68.5 ^a^	0.0001
Concentrate (kg)	460.3 ± 305.5 ^a^	319.4 ± 156.5 ^b^	202.4 ± 138.0 ^b^	202.4 ± 138.0 ^b^	0.0001
Electricity consumption (E/sheep)	15.9047 ± 16.82	24.18 ± 19.95	23.71 ± 14.42	23.71 ± 14.42	0.3500
Fuel use (E/sheep)	9.92 ± 10.02	9.75 ± 7.32	10.63 ± 8.92	10.63 ± 8.92	0.9200
Number of animal species	1.17 ± 0.38	1.63 ± 0.96	1.43 ± 0.69	1.43 ± 0.69	0.2100
Native animals (%)	0.0 ± 0.0 ^c^	55.47 ± 49.79 ^b^	100.00 ± 0.00 ^a^	100.00 ± 0.00 ^a^	0.0001
Minerals (kg)	11.46 ± 13.13	63.13 ± 117.28	23.98 ± 52.28	23.98 ± 52.28	0.1100
Transformation into cheese (%)	0.0 ± 0.0	6.03 ± 21.7	13.06 ± 32.83	13.06 ± 32.83	0.4200
FWU/WU	0.89 ± 0.23	0.74 ± 0.26	0.84 ± 0.27	0.84 ± 0.27	0.1900

^abc^ Different letters in the same row are statistically different (*p* < 0.05). kg of concentrate purchased per animal per year, hectares per useful agricultural area per sheep (AAS ha/animal), minerals that have been added to the land (kg of minerals/ha), relationship between family work unit and work unit (FWU/WU).

**Table 2:** Attributes, critical points, and indicators selected in the case studied.

Attributes	Critical Points	Diagnostic Criterion	Indicator	Unit	Dimension
Productivity	Inadequate management of resource use	Efficiency	Milk per sheep	kg/animal	Profitability (economy)
Productivity	Milk storage (must be kept refrigerated at 4 °C), ripening room, and cheese making	Efficiency	Transformation into cheese	%	Profitability (economy)
Productivity	Inadequate management of resource use	Efficiency	Lambing per year	No	Planet (environmental)
Productivity	Inadequate management of resource use	Intensification	Animals in housing	%	Planet (environmental)
Productivity	Inadequate management of resource use	Intensification	Number of sheep	No	Planet (environmental)
Productivity	High dependency on external resources	Intensification	Relationship between family work unit and work unit	FWU/WU	People (social)
Stability, reliability, and resilience	Grassland area	Grassland area	AAS ha/sheep	Ha/animal	Planet (environmental)
Stability, reliability, and resilience	Grassland area	Intensification	Communal land use	Days/animal	Planet (environmental)
Stability, reliability, and resilience	High dependency on external resources	Intensification	Concentrate	kg	Profitability (economy)
Adaptability	High dependency on external resources	Intensification	Electricity consumption (E/ewe)	Kw/ewe	Profitability (economy)
Adaptability	High dependency on external resources	Intensification	Fuel use (E/ewe)	L/ewe	Profitability (economy)
Stability, reliability, and resilience	High dependency on external resources	Intensification	Minerals	kg/ha	Planet (environmental)
Stability, reliability, and resilience	Low biological diversity	Intensification	Number of animal species	No	Planet (environmental)
Stability, reliability, and resilience	Low biological diversity	Breeds	Native animals	%	Planet (environmental)

**Table 3:** Definition of factors, integration of variables, and percentage of total variance that contributed to the total variance in dairy sheep production systems in different regions of Castilla y León, Spain.

Factor	Name	Variable(s)	Percentage	Percentage Accumulated
F1	Milk production	kg milk/sheep/year, standard milk, kg of concentrate purchased per animal per year, stabling, communal days/animal per year, and percentage of native animals of the total herd	23.8	23.8
F2	Animal species in the herd	Animal species present in the herd: number of animal species present in the herd	18.1	41.9
F3	Product transformation	Size of the herd or number of animals (sheep), births per year, if there is transformation of cheese, and the family work unit relationship (FWU/WU)	14.2	56.1
F4	Energy use	Use of energy: electricity/animal consumption per year, use of fuel/sheep per year, and kilograms of minerals/ha added to the land.	8.8	64.9
F5	Hectares for livestock	Hectares per useful agricultural area per sheep	7.2	72.1

Hectares per useful agricultural area per sheep (AAS ha/animal), relationship between family work unit and work unit (FWU/WU).

**Table 4:** Mean values of sheep and goat production systems according to production unit groups in different regions in Spain.

Variable	PU1 Semi-Extensive Dairy Systems	PU2 Semi-Intensive Systems	PU3 Semi-Extensive Cheese Systems	PU4 Intensive Systems	*p*-Value
Number of farms	14	34	4	18	
Herd size	305.0 ± 71.0 ^b^	394.0 ± 38.0 ^b^	898.0 ± 117.0 ^a^	720.0 ± 570.0 ^a^	0.0001
Births per year	1.91 ± 0.20 ^b^	1.41 ± 0.11 ^b^	3.75 ± 0.34 ^a^	3.08 ± 0.16 ^a^	0.0001
kg of milk per sheep per year	307.0 ± 28.0 ^a^	157.0 ± 15.0 ^b^	63.0 ± 46.0 ^b^	287.0 ± 23.0 ^a^	0.0001
kg of concentrate purchased	353.0 ± 55.0 ^ab^	250.0 ± 30.0 ^bc^	64.0 ± 92.0 ^c^	418.0 ± 45.0 ^a^	0.0020
Housing system	3.7 ± 3.0 ^c^	36.7 ± 3.2 ^b^	23.9 ± 9.9 ^bc^	82.8 ± 4.8 ^a^	0.0001
AAS ha/animal	0.45 ± 0.05 ^a^	0.16 ± 0.03 ^b^	0.21 ± 0.09 ^ab^	0.09 ± 0.04 ^b^	0.0001
Community days per animal per year	71.0 ± 16.2 ^b^	77.0 ± 8.7 ^b^	127.0 ± 26.8 ^a^	6.08 ± 13.0 ^b^	0.0001
Electricity consumption per animal per year	3.6 ± 4.7 ^b^	27.6 ± 2.5 ^b^	25.8 ± 7.8 ^ab^	23.0 ± 3.8 ^a^	0.0001
Fuel per sheep per year	4.9 ± 2.5	11.2 ± 1.3	10.3 ± 4.2	10.9 ± 2.0	0.1700
Other species	2.7 ± 0.18 ^a^	1.4 ± 0.10 ^b^	1.0 ± 0.30 ^b^	1.0 ± 0.14 ^b^	0.0001
Percentage of native animals of the total herd	78.8 ^a^ ± 6.7	93.4 ^a^ ± 3.6	100.0 ^a^ ± 11.1	3.8 ^b^ ± 5.4	0.0001
kg of minerals/ha	234.2 ± 25.3	59.9 ± 13.6	391.0 ± 42.0	23.6 ± 20.4	0.1300
Transformation to cheese	3.5 ± 0.7 ^b^	1.7 ± 0.4 ^b^	100.0 ± 1.22 ^a^	0.0 ± 0.0 ^c^	0.0001
FWU/WU	0.72 ± 0.08	0.83 ± 0.04	0.71 ± 0.13	0.84 ± 0.05	0.4900

^abc^ Different letters in the same row are statistically different (*p* < 0.05). Production units (PUs), kg of concentrate purchased per animal per year, hectares per useful agricultural area per sheep (AAS ha/animal), minerals that have been added to the land (kg of minerals/ha), relationship between family work unit and work unit (FWU/WU).

## Data Availability

The data used to support the findings of this study are available from the corresponding author upon reasonable request.
